# Correlation between Cortical State and Locus Coeruleus Activity: Implications for Sensory Coding in Rat Barrel Cortex

**DOI:** 10.3389/fncir.2016.00014

**Published:** 2016-03-24

**Authors:** Zeinab Fazlali, Yadollah Ranjbar-Slamloo, Mehdi Adibi, Ehsan Arabzadeh

**Affiliations:** ^1^School of Cognitive Sciences, Institute for Research in Fundamental Sciences (IPM)Tehran, Iran; ^2^Eccles Institute of Neuroscience, John Curtin School of Medical Research, The Australian National UniversityCanberra, ACT, Australia; ^3^Australian Research Council Centre of Excellence for Integrative Brain Function, The Australian National University NodeCanberra, ACT, Australia

**Keywords:** cortical state, synchronized, desynchronized, locus coeruleus, neuromodulation, vibrissal system, somatosensory cortex

## Abstract

Cortical state modulates the background activity of cortical neurons, and their evoked response to sensory stimulation. Multiple mechanisms are involved in switching between cortical states including various neuromodulatory systems. Locus Coeruleus (LC) is one of the major neuromodulatory nuclei in the brainstem with widespread projections throughout the brain and modulates the activity of cells and networks. Here, we quantified the link between the LC spontaneous activity, cortical state and sensory processing in the rat vibrissal somatosensory “barrel” cortex (BC). We simultaneously recorded unit activity from LC and BC along with prefrontal electroencephalogram (EEG) while presenting brief whisker deflections under urethane anesthesia. The ratio of low to high frequency components of EEG (referred to as the L/H ratio) was employed to identify cortical state. We found that the spontaneous activity of LC units exhibited a negative correlation with the L/H ratio. Cross-correlation analysis revealed that changes in LC firing preceded changes in the cortical state: the correlation of the LC firing profile with the L/H ratio was maximal at an average lag of −1.2 s. We further quantified BC neuronal responses to whisker stimulation during the synchronized and desynchronized states. In the desynchronized state, BC neurons showed lower stimulus detection threshold, higher response fidelity, and shorter response latency. The most prominent change was observed in the late phase of BC evoked activity (100–400 ms post stimulus onset): almost every BC unit exhibited a greater late response during the desynchronized state. Categorization of the BC evoked responses based on LC activity (into high and low LC discharge rates) resulted in highly similar response profiles compared to categorization based on the cortical state (low and high L/H ratios). These findings provide evidence for the involvement of the LC neuromodulatory system in desynchronization of cortical state and the consequent enhancement of sensory coding efficiency.

## Introduction

In order to adapt to various environmental and behavioral demands, the brain switches between multiple modes of processing. Brain state modulates the background activity of cortical neurons (Castro-Alamancos and Oldford, 2002; Gentet et al., 2010, 2012; Hirata and Castro-Alamancos, 2011; Sakata and Harris, 2012; Polack et al., 2013; Reimer et al., 2014), and their response profile to sensory stimulation (Castro-Alamancos and Oldford, 2002; Castro-Alamancos, 2004; Murakami et al., 2005; Crochet and Petersen, 2006; Niell and Stryker, 2010; Polack et al., 2013; Sellers et al., 2013; Zagha et al., 2013; Lee et al., 2014; McGinley et al., 2015b; Vinck et al., 2015), and thus affects the transmission of information along the sensory pathway. Although cortical state changes along a continuum (Harris and Thiele, 2011; Zagha and McCormick, 2014), two distinct modes are identified at the global and cellular levels: synchronized and desynchronized states. These states are identifiable based on the fluctuation profile of membrane potential (V_m_) of single neurons, the local field potentials (LFP), and the global electroencephalogram (EEG) signals. Due to the synchronous activity of neuronal populations, the synchronized state is dominated by slow-wave oscillations (<4 Hz) while the desynchronized state lacks such prominent slow oscillations (Harris and Thiele, 2011). The synchronized state is associated with slow-wave sleep and quiet waking whereas the desynchronized state is associated with active waking and rapid eye movement (REM) sleep (Steriade et al., 1993; Poulet and Petersen, 2008; Lee and Dan, 2012; McCormick et al., 2015). However, both states are observed during anesthesia in rodents and primates (Murakami et al., 2005; Clement et al., 2008; Cheong et al., 2011; Bermudez Contreras et al., 2013; Pachitariu et al., 2015). Multiple mechanisms are suggested to be involved in the switches between the cortical states including thalamic input (Hirata and Castro-Alamancos, 2010; Poulet et al., 2012), motor cortex feedback (Zagha et al., 2013) and the neuromodulatory systems (Lee and Dan, 2012; Sara and Bouret, 2012; Eggermann et al., 2014; Zagha and McCormick, 2014).

Neuromodulatory systems can alter network activity and cortical state during sleep-wake cycle, arousal, attention and stress (Li et al., 2009; Lee and Dan, 2012; Sara and Bouret, 2012; Eggermann et al., 2014). Locus Coeruleus (LC), the principal nucleus in the brainstem releasing the neuromodulator norepinephrine (NE), has widespread projections throughout the brain (Cedarbaum and Aghajanian, 1978; Foote et al., 1983; Aston-Jones et al., 1986; Szabadi, 2013; Schwarz and Luo, 2015). LC activity is associated with level of arousal, sleep-wake cycle and behavioral states (Aston-Jones et al., 2001; Samuels and Szabadi, 2008). Higher levels of activity in LC correspond to higher levels of arousal (Rajkowski et al., 1994; Berridge, 2008; Carter et al., 2010; Vazey and Aston-Jones, 2014), the transition from sleep to waking (Aston-Jones and Bloom, 1981a), and active engagement in a behavioral task (Foote et al., 1980; Aston Jones, 1985). Perturbations of LC activity or NE receptors in the cortex also affect cortical state (Berridge and Foote, 1991; Berridge et al., 1993; Carter et al., 2010; Constantinople and Bruno, 2011; Polack et al., 2013; Castro-Alamancos and Gulati, 2014). Electrical micro-stimulation of LC (Bouret and Sara, 2002; Berridge and Waterhouse, 2003; Devilbiss and Waterhouse, 2004, 2011; Lecas, 2004; Devilbiss et al., 2006; Sara, 2009) or the administration of NE to sensory areas (Kössl and Vater, 1989; McCormick, 1989; McCormick et al., 1991; Devilbiss and Waterhouse, 2000; Waterhouse et al., 2000; Berridge and Waterhouse, 2003; Hurley et al., 2004) affect sensory processing across different modalities.

Here, we focused on the spontaneous discharge of LC neurons during prolonged recordings, and quantify the extent to which the spontaneous activity of LC correlates with the cortical state, and affects the transmission of sensory information. The rodent vibrissal area of the somatosensory cortex, also known as the barrel cortex (BC) is a well-established model of cortical processing with an elegant cortical organization and high level of functional efficiency (Brecht et al., 1997; Petersen, 2007; Kleinfeld and Deschênes, 2011; Diamond and Arabzadeh, 2013; Feldmeyer et al., 2013).

## Materials and Methods

### Surgery and Electrophysiological Recording

Twenty-four adult male Wistar rats, weighing 300–390 g were used. All experiments were approved by the animal care and experimentation committee of the Institute for Research in Fundamental Sciences (IPM). Anesthesia was induced by intra-peritoneal administration of urethane (1.5 g/Kg), was monitored by hind paw and corneal reflexes and maintained stable with supplemental doses of urethane (0.1 g/Kg) if necessary. Body temperature was maintained at 37°C by a heating blanket (Harvard Apparatus, Holliston, MA, USA). Two craniotomies were performed on the right hemisphere to provide access to BC (5 × 5 mm; centered at 2.6 mm posterior and 5 mm lateral to Bregma) and LC (4 × 4 mm; centered at 10.8 mm posterior to Bregma and 1.4 mm lateral to the midline). To facilitate the access to LC, the animal’s head was tilted down by about 14° (Bouret and Sara, 2002).

**Figure 1:**
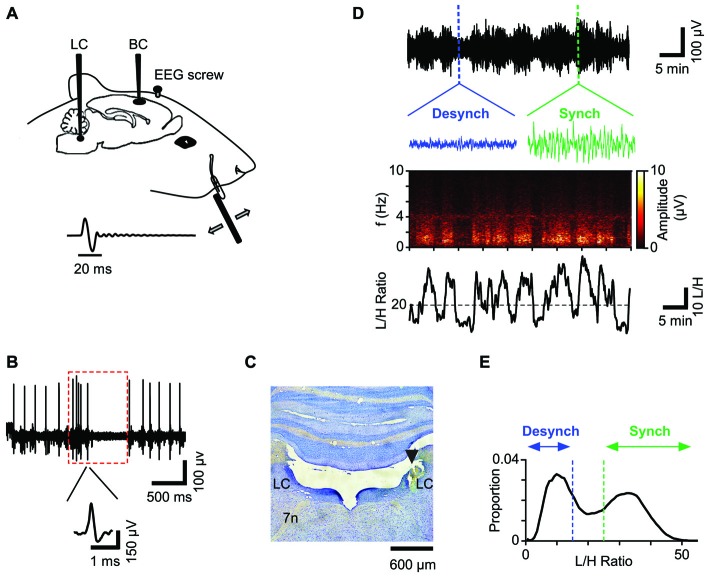
**Schematic illustration of the recording paradigm. (A)** Unit activity was recorded from Barrel Cortex (BC) and Locus Coeruleus (LC) while simultaneously recording EEG from prefrontal area. For sensory stimulation a full-cycle sinusoidal deflection was applied to the contralateral whisker. **(B)** Spiking activity of a representative LC neuron. Red box represents typical response of a single LC neuron to a paw pinch: the response shows a biphasic characteristic with a brief high frequency spiking followed by a longer suppression. Inset shows the neuron’s spike waveform. **(C)** Histological verification of the LC recording site for one session. **(D)** The low-pass filtered EEG from a typical recording session (upper panel) and the corresponding spectrogram (middle panel) reveal high amplitudes at low frequency bands (<4 Hz) during the synchronized state. Insets show 12-s traces of the synchronized state (green) and the desynchronized state (blue). The lower panel plots the corresponding L/H ratio: the ratio of amplitudes in Low (0.5–4 Hz) to High (20–60 Hz) frequency ranges. **(E)** Distribution of L/H ratios for the same session shows a biphasic profile corresponding to the two states; the synchronized state (green) and the desynchronized state (blue). This color convention will be used henceforth.

We simultaneously recorded neuronal activity in LC and BC along with the prefrontal EEG (Figure 1A). Data acquisition and online amplification were performed using a NikTek recording system (NikTek, Tehran, Iran). BC neuronal activity was acquired with single tungsten microelectrodes (1–2 MΩ, FHC Inc., ME, USA). The principal whisker was determined by manual stimulation of individual whiskers. The recordings were made from 650 to 1400 μm from surface of the exposed dura (*n* = 27). The onset response latency of <7 ms and the median depth of 850 μm, suggest that the recorded neurons were mostly located in layer four BC. Spiking activity of LC (ipsilateral to BC) was obtained by single tungsten microelectrodes (0.5–1 MΩ, FHC Inc., ME, USA) from 5.6 to 5.9 mm below the dura. To confirm the recording site, we used the following criteria (Figure 1B): LC neurons usually have wide extracellular spike waveforms (>0.6 ms), and respond to paw pinch with a typical excitation-inhibition pattern (red box in Figure 1B; Cedarbaum and Aghajanian, 1978). The spiking activity in response to the paw pinch was monitored via a loudspeaker and visualized on a digital oscilloscope for examination of the spiking profile. For well-isolated single units (Figure 1B) the firing rate was low (0.1–6 Hz) consistent with previous literature. Additionally, at the end of the experiment, we further verified the LC recording site by histology (Figure 1C). EEG recordings were obtained from a stainless steel screw placed above the prefrontal cortex (2–4 mm anterior to Bregma and 0.5 mm lateral to the midline) with the reference electrode attached to a second screw implanted above the cerebellum.

Neuronal data were recorded at a sampling rate of 30 kHz and filtered on-line by applying a band-pass filter (300–6000 Hz) for spiking activity. Spikes were extracted by off-line sorting using principal component analysis implemented in MATLAB (Math Works). For multi-unit recordings from LC, we set a liberal threshold for spike detection. The multi-unit firing rates (range 7.9–94.1 spikes/s, median: 32.4) were thus higher than the typical single-unit firing rates expected from LC (0.1–6 Hz) (Cedarbaum and Aghajanian, 1978). Similarly for BC multi-units, we set a liberal threshold for spike inclusion (range 0.9–96.2 spikes/s, median = 18.8). For cross correlation analysis, we used multi-unit activity. However for BC sensory evoked analysis, we sorted single-units as well as multi-units. Spontaneous firing rates of BC single-units varied between 0.5 and 28.6 spikes/s (median = 3.1). In total, 63 units were extracted from BC recordings (27 single- and 36 multi-units) and 34 multi-units were extracted from LC recordings.

### Stimulus Presentation

Single cycle 80 Hz sinusoidal deflections were delivered to the BC neuron’s principal whisker using a piezoelectric device. The principal whisker was placed into the microelectrode with a 2 mm distance from the base of the whisker. We used an infrared optic sensor to calibrate the piezo movement range and confirmed that it accurately followed the voltage command (Figure 1A). For the range of stimulus intensities applied here (amplitudes: 6–60 μm), the post-deflection resonance was negligible (<6% of the maximum amplitude) and was not detectable beyond 70–80 ms. To optimize the stimulation amplitudes based on the dynamic range of a unit, we adjusted the stimulation intensity for each BC unit based on its response threshold (see below). This adjustment was performed at the beginning of each recording session by applying 10 levels of deflection from a relatively wide range of amplitudes (0–54 μm with 6 μm steps, 50 repetitions each). A Nuka-Rushton function was fitted to the average spike count to characterize the neuronal response function. The threshold (T; 12–30 μm) was defined as the inflection point of this function—i.e., the stimulus amplitude that produced half of the maximum response dynamic (M_50_, Adibi et al., 2013). The main recording protocol lasted 120 min. This included recording of spontaneous cortical activity and the evoked response to a set of amplitudes ($\frac{1}{2}$T, T, 1$\frac{1}{2}$T, and 2T) presented in a pseudorandom order with an inter-stimulus interval of 5 s (26 sessions) or 10 s (5 sessions). This long recording ensured that each stimulus was repeated in each session for a sufficient number of trials during both synchronized (mean number of trials 73.3) and desynchronized states (104.7).

### EEG Analyses

Prefrontal EEG and BC LFP signals were filtered off-line between 0.1 and 100 Hz. Amplitude spectra were computed in MATLAB using Fast Fourier Transform (FFT) applied to 2s non-overlapping sliding windows. In previous studies, the frequency components of EEG or LFP have been used as an indicator of cortical state (Clement et al., 2008; Poulet and Petersen, 2008; Goard and Dan, 2009; Li et al., 2009; Polack et al., 2013; Lee et al., 2014). Similarly, here, we defined the average amplitude of low (0.5–4 Hz) to high frequencies (20–60 Hz) (Low/High, L/H ratio) as the index of cortical state as previously used (Li et al., 2009). We also calculated the L/H ratio based on the BC LFP signal, which produced qualitatively similar results to that of the prefrontal EEG. However, the difference between the synchronized and desynchronized states was more evident in the prefrontal EEG. Another advantage of the prefrontal EEG signal was that, unlike BC LFP, it did not contain any sensory evoked components. Throughout the article, L/H ratio thus refers to the classification based on the prefrontal EEG. The L/H ratio was normalized (*z*-scored) for each recording session by subtracting the average within-session L/H ratio and dividing by the within-session standard deviation.

### Spontaneous Neuronal Activity

**Figure 2:**
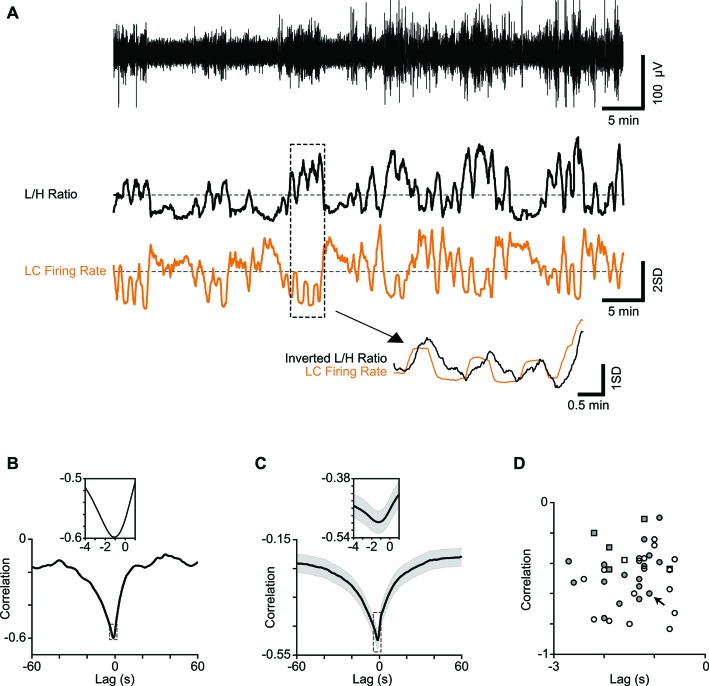
**Correlation between LC firing rate and cortical state. (A)** One-hour low-pass filtered EEG from a typical recording session (upper trace) and the corresponding L/H ratio (middle trace) aligned with the simultaneously recorded LC firing rate (orange). Both traces are *z*-scored. Four minutes of recording are magnified below to illustrate the temporal relation in more detail. Note the L/H ratio is inverted in the magnified trace to better visualize the relative timings. **(B)** Cross-correlation of a representative session in **(A)** shows maximum correlation at −1.1 s. The inset shows a 5-s window around maximum correlation. **(C)** Average cross correlation across 34 sessions (multi-units). The inset shows a 5-s window around maximum correlation. **(D)** Every dot represents one session. Squares represent single-units and circles represent multi-units. Filled symbols show units for which the electrode position in LC was histologically confirmed. The arrow shows the sample session in **(A)** and **(B)**.

To isolate the spontaneous activity from the evoked response to whisker deflections, we removed a 1s window after every stimulus presentation from the BC, LC and the L/H ratio time series. Firing rate of LC and BC were calculated with a 2s bin and then *z*-scored over time before measuring the Pearson’s correlation coefficient (Figures 2A,D). To quantify the temporal dynamics of correlations, we also calculated cross-correlations at incremental lags of 100 ms (Figures 2B–D). For 28/36 sessions, the distribution of the L/H ratios showed a bimodal profile (as illustrated in Figure 1E). This allowed us to define two boundaries on the L/H ratio to allocate spontaneous activity into episodes of synchronized and desynchronized state. In 8 out of 36 recording sessions, a bimodal distribution was not evident. For these sessions, we assigned the lower and higher thirds of the L/H ratios to desynchronized and synchronized states.

### Stimulus Evoked Response

To compare the response characteristics in the two states, we first categorized trials based on their L/H ratio during a 5-s window around the stimulus onset. Trials were categorized into synchronized and desynchronized based on their corresponding L/H ratio value on the L/H distribution (Figure 1E). Early and late neuronal responses were defined as spike counts over the windows 0–50 and 100–400 ms post stimulus onset, respectively. The trial-to-trial response variability was estimated in terms of the Fano factor: variance divided by the mean of early responses across trials.

For spike time analysis, response delay was determined as the time of the first post-stimulus bin (0.5 ms) that exceeded 3× standard deviation of the baseline activity (average of a 500ms window before stimulus onset).

### ROC Analyses

To quantify stimulus detectability, we used a receiver operating characteristic (ROC) analysis (Green and Swets, 1966). The AUROC provides an index of neuronal performance for stimulus detection taking into account the trial-to-trial variability in response. To calculate AUROC for each state, spike counts were used to create signal distribution while corresponding spike counts of the baseline activity (a 50-ms window before the stimulus onset) were used to create noise distribution. All possible values of the decision criterion, ranging from the minimum to the maximum observed spike counts were used to calculate hit rate (the fraction of signal distribution above criterion) and false-alarm rate (the fraction of noise distribution above criterion). The profile of the hit rates vs. false alarm rates defines the ROC curve. We used the trapezoid method to calculate the AUROC. The AUROC was calculated for a 50-ms sliding window from −100 to 400 ms post stimulus onset.

### Gaussian Mixture Model and d-prime Analyses

For the joint distribution of LC firing rate and L/H ratio, we applied a bivariate Gaussian mixture model comprised of two bivariate Gaussian components with the 2 × 1 mean vectors *μ*_1_ and *μ*_2_, and 2 × 2 covariance matrices C_1_ and C_2_. The distance between these two components was quantified in terms of the *d*^2^

$$d^{\text{2}}\;\text{=}\;{\left(\mu_{\text{1}}\text{-}\mu_{\text{2}}\right)}^{\text{T}}\times \;{\text{C}}^{-1}\times (\mu_{\text{1}}\text{-}\mu_{\text{2}})$$

where ^T^ denotes the matrix transpose operation and C represents the average covariance matrix defined as $\frac{1}{2}$(C_1_ + C_2_). This measure is related to the Mahalanobis and Bhattacharyya distance (Mahalanobis, 1936; Bhattacharyya, 1946).

To calculate the separation along each of the two dimensions (LC firing rate, and L/H ratio) the data points were projected onto that dimension and the distance, *d*^′^, was calculated based on the following equation:

$$d^{\prime }\;=\;\frac{\left(\mu_{\text{1}}\text{-}\mu_{\text{2}}\right)}{\sqrt{\frac{1}{2}(\sigma_{1}{}^{2}+\sigma_{2}{}^{2})}}$$

where *μ*_1_, *μ*_2_ and *σ*_1_^2^, *σ*_2_^2^ denote the means and variances of the marginal Gaussian components respectively. This *d*^′^ is the special case of the squared root of *d*^2^ along one dimension.

### Statistical Analyses

For statistical comparison of the difference between the mean values of two given groups, we performed random permutation tests, unless otherwise indicated. We randomly shuffled the samples between the two groups and re-calculated the difference in the means for the shuffled data. This procedure was repeated 1000 times and a distribution of differences (null distribution) was obtained. The null hypothesis was tested against the observed difference with false-rejection probabilities of *α* = 0.05, 0.01, and 0.001.

### Histology

At the end of the experiment, an electrical lesion was made by passing a DC current at 9 V through the LC electrode tip for 10 s. After transcardial perfusion with ~300 ml saline (0.9%) followed by ~300 ml phosphate-buffered formalin (10%, pH = 7.4), the brain (*n* = 10) was removed and kept in formalin (for a minimum of 1 week) before 10 μm thick coronal sections were made. Sections were Nissl stained and lesions were detected by light microscopy. LC location was compared with the lesion site using the rat brain atlas (Paxinos and Watson, 2007). Although the lesion was often larger than LC, its center was at the position of LC in the atlas. This confirmed reliability of our electrophysiological criteria. The main findings remained unchanged when we limited the analysis to units that were recorded during the histologically verified sessions (10 rats).

## Results

We simultaneously recorded neuronal activity from BC and LC along with the prefrontal EEG in urethane anesthetized rats (Figure 1A). This allowed us to quantify the interaction between three parameters: (i) sensory representation in a primary sensory cortex (BC neuronal activity); (ii) neuromodulatory activity (LC neuronal activity); and (iii) cortical state (as identified by prefrontal EEG). LC recording was confirmed based on broad spike waveforms (>0.6 ms), the typical response profile to noxious stimulation (Figure 1B) and histology (Figure 1C). BC recording was confirmed based on the neuronal response to brief deflections (single-cycle sinusoidal vibration, 12.5 ms duration) applied to the neuron’s principal whisker. This initial stimulation, additionally, allowed us to estimate the neuron’s response threshold (T) using a wide range of deflection amplitudes (see “Materials and Methods” Section). The main recording protocol included long durations of spontaneous activity, as well as periods when single deflections were applied to the BC neuron’s principal whisker. The deflection amplitudes were adjusted for each neuron based on the initial estimation of the neuronal response function: the amplitudes were 0, $\frac{1}{2}$T, T, 1$\frac{1}{2}$T, and 2T.

### Cortical State

During each recording session, the prefrontal EEG amplitude alternated between two patterns of activity (Figure 1D), known as the synchronized and desynchronized states. The synchronized state was identified by high-amplitude low-frequency (<4 Hz) oscillations, which were absent in the desynchronized state. These patterns could be distinguished by the Fourier transformation of the EEG signal: in each session, the average amplitude of low (0.5–4 Hz) to high frequencies (20–60 Hz) (Low/High, referred to as the L/H ratio) reliably captured the temporal fluctuations in the cortical state (Figure 1D). Two boundaries were determined for each session based on the bimodal distribution of L/H ratios for synchronized and desynchronized states (Figure 1E; see “Materials and Methods” Section).

### Fluctuations of LC Firing Rate Precede Fluctuations of Cortical State

To establish the link between cortical state and LC neuronal activity, we quantified the temporal profile of firing for LC units, along with the temporal profile of the simultaneously calculated L/H ratio. Figure 2A illustrates these time series for an example recording epoch revealing the anti-correlation between the two traces. This pattern of anti-correlation was representative of all recorded units: for all 34 recording sessions, every LC single and multi-unit showed a negative correlation with the L/H ratio (all p values < 0.01) with an average correlation coefficient of −0.28 ± 0.05 and −0.46 ± 0.03, respectively (*n* = 6 and *n* = 34; mean ± SEM). To characterize the temporal dynamics of the correlation between LC firing and L/H ratio, we quantified the cross-correlation between the two traces with temporal lags advancing at 0.1 s steps (see “Materials and Methods” Section). Figure 2B illustrates the cross-correlation between LC firing rate and L/H ratio for the example recording session in Figure 2A. For this recording session, the strongest correlation was at −1.1 s time lag (Figure 2B), revealing that fluctuations in LC firing rate precede fluctuations in L/H ratio. This finding was replicated across recording sessions: the cross-correlogram showed a trough at an average time lag of −1.21 ± 0.1 s (and an average correlation of −0.50 ± 0.03, Figure 2C). A similar result was found for the six well-isolated single-units (Figure 2D). Figure 2D plots for each of the recorded units the strongest correlation value against its corresponding time lag. For all recorded units, the strongest correlation occurred at a negative lag (Figure 2D).

### Relation between L/H Ratio and BC Firing Rate

We examined whether the strong relation between LC firing rate and cortical state was specific to LC neurons or generalized to other neurons such as those recorded from sensory cortex. We quantified the temporal profile of firing in BC units along with the temporal profile of the simultaneous L/H ratio. In contrast to LC, BC neurons did not show a systematic relation with cortical state: across 27 single-units, 16 units were positively correlated (average correlation of 0.26 ± 0.03, mean ± SEM, *p* < 0.05), four units were negatively correlated (−0.34 ± 0.10, *p* < 0.05), and seven units did not show a significant correlation. Across 36 multi-units, 21 units showed significant positive correlation (0.21 ± 0.03, *p* < 0.05), 9 units showed significant negative correlation (−0.26 ± 0.07, *p* < 0.05), and 6 units did not show a significant correlation. This diversity is consistent with previous recordings of cortical cells demonstrating that state modulates various cell-types differently (Castro-Alamancos and Oldford, 2002; Gentet et al., 2010, 2012; Hirata and Castro-Alamancos, 2011; Sakata and Harris, 2012; Polack et al., 2013; Reimer et al., 2014). In our data, the diversity in BC-L/H correlation could not be explained by the depth of recording (*p* = 0.40) or by the activity of the simultaneously recorded LC neuron.

### Effect of Cortical State on the Spontaneous Activity of BC and LC

To determine how cortical state affected the background activity of LC and BC units, we identified the state of any given time instance based on the value of L/H ratio at that time instance. Across all sessions, this method classified 26 ± 2% of the total recording duration as synchronized state and 39 ± 2% as desynchronized state. LC units consistently fired more spikes during the desynchronized state compared to the synchronized state (signed rank test, *p* < 0.001).

Consistent with the observed diversity in BC-L/H correlations, BC firing rates did not show a systematic dependency on the state: 59% of units exhibited higher firing during the synchronized state while others showed either little difference (22%) or a higher firing during the desynchronized state (19%).

### Effect of Cortical State on Early and Late Sensory-Evoked Responses

**Figure 3:**
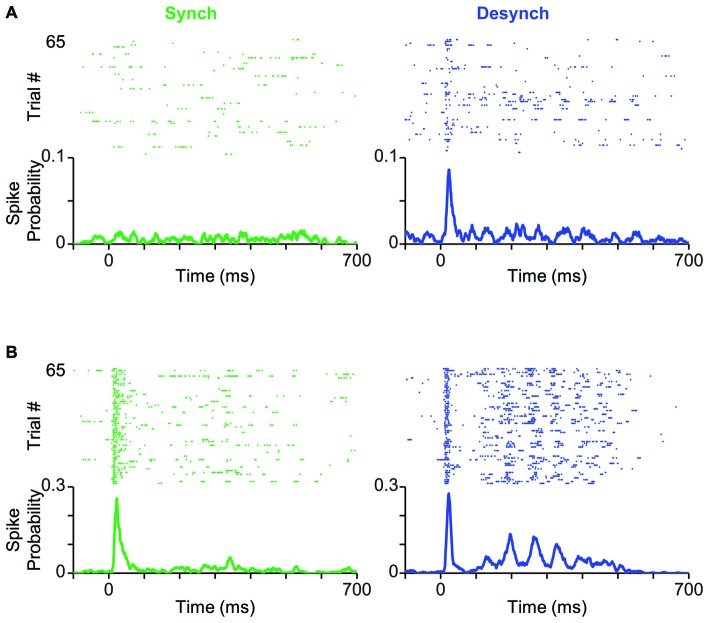
**Cortical state modulates response profile of BC units. (A)** Neuronal activity of a typical BC single-unit in response to the threshold-level stimulus amplitude (T; 12 μm). Trials are separated based on the cortical state (synchronized in green, desynchronized in blue) and aligned to the stimulus onset (0 ms). PSTHs show the spike probability across trials at each time bin (bin size = 1 ms). **(B)** Same as **(A)** but for the highest stimulus amplitude (2T; 24 μm).

How does cortical state modulate the response profile of BC neurons to whisker stimulation? To address this question, we classified trials into synchronized and desynchronized states based on their corresponding L/H ratio. Figure 3A shows a BC unit, which exhibited prominent state-dependent modulation of its early response to a 12 μm deflection (T). Trials are separated based on their state: synchronized (left panel in green) or desynchronized (right panel in blue). This neuron produced a stimulus-evoked response that was significantly above its background firing (Wilcoxon rank-sum test, *p* < 0.05) only in the desynchronized state. A similar trend was observed for 11 out of 55 units (20%), where the low-intensity deflection produced significant response (Wilcoxon rank-sum test, *p* < 0.05) only in the desynchronized state. Four out of 55 units (7%) showed the opposite trend of significant responses only in the synchronized state. Figure 3B shows response of the same unit as in Figure 3A to repeated presentations of a 24 μm stimulus (2T). The unit fired greater number of spikes in the desynchronized state compared to the synchronized state. This modulation was most prominent in the late phase of the response (from 100 to 400 ms post stimulus onset). This modulation of the late response for the highest amplitude was consistent across 88% of recorded units (see below).

### Effect of Cortical State on Stimulus-Response Function

**Figure 4:**
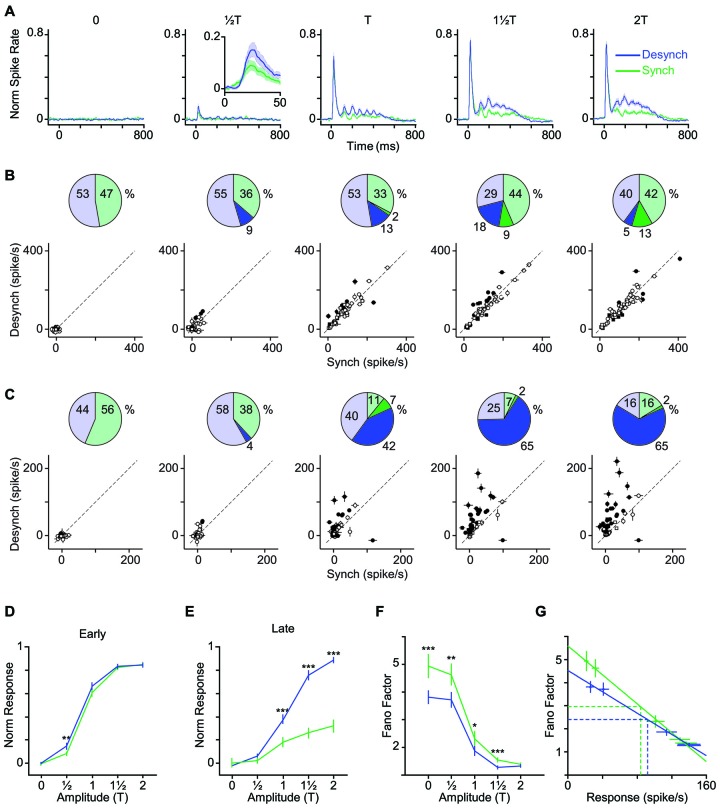
**Modulation of early and late response by cortical state. (A)** The average neuronal response across all recorded units to the full range of stimulus amplitudes. PSTHs are generated with a 10-ms bin size. Shaded error bars represent SEM across units (*n* = 55) (for each session, PSTHs are normalized to the maximum spike per bin across amplitudes and states). Inset for $\frac{1}{2}$T shows a 50-ms window of response.** (B)** Scatter plot of the early phase of evoked response (0–50 ms after stimulus onset) in synchronized vs. desynchronized states. Squares represent single-units and circles represent multi-units. Filled symbols show units with significant difference between the two states (random permutation tests, *p* < 0.05). The pie plots show the percent of units that produced higher response in the desynchronized (blue) and synchronized states (green). Darker blue and green parts show percent of units with significant response change in each state (percentages are indicated outside the circles). **(C)** Same as in **(B)** but for the late phase of evoked response (100–400 ms after stimulus onset). Average baseline activity (500 ms window before stimulus onset) was subtracted from the response. **(D)** Average response function across all recorded BC units. Neuronal activity was defined as the early response (spike count in 0–50 ms post stimulus onset). Error bars are SEM across units (*n* = 55). **(E)** Same as **(D)** but for late response (spike count in 100–400 ms post stimulus onset). ***p < 0.001, and **p < 0.01, statistical significance based on random permutation test. For every unit, the average baseline activity (500 ms window before stimulus onset) was subtracted from the response. **(F)** Fano factor as a function of the stimulus amplitude. Error bars are SEM Fano factor across units. (***p < 0.001, **p < 0.01, and *p < 0.05, random permutation test). **(G)** Fano factor as a function of mean response across all units. Each data point represents one state-amplitude combination. Horizontal and vertical dashed lines show the mean of average values along *y*- and *x*-axes for each state. Spike count is calculated over a 50-ms window post stimulus onset. Solid lines show fitted functions to each state’s data points. Vertical error bars are SEM Fano factor across units. Horizontal error bars are SEM spike rate across units.

How does cortical state modulate the neuronal response across the range of stimulus intensities? Figure 4A illustrates the PSTH averaged across all recorded units at each level of stimulation. Figures 4B,C quantify the change in the early and late phases of response across stimulus intensities for each of the recorded units. Although some units showed significant modulation of their early response by state (filled circles; *p* < 0.05, random permutation test followed by correction for multiple comparisons based on the Benjamini-Hochberg procedure), this modulation was not systematic across all units (Figure 4B, pie charts). Furthermore, modulation of response by state was highly dependent on stimulus intensity. At low amplitudes ($\frac{1}{2}$T, T), 65% of units elicited an early response that was higher in the desynchronized state, but this trend disappeared at higher amplitudes (Figure 4B).

Unlike the early response, the late response was systematically modulated by state across neurons and stimulus intensities. When stimulated with amplitudes at or above their response threshold (T, 1$\frac{1}{2}$T and 2T), the majority of units (82%) elicited a late response that was higher in the desynchronized state (Figure 4C).

Figures 4D,E show the stimulus response function for the population, quantified separately for early and late phases of the response. Across neurons, the modulation of early response was significant only at $\frac{1}{2}$T (Figure 4D), although a similar trend existed for the T and 1$\frac{1}{2}$T amplitudes. The late phase of response (Figure 4E) showed a gain modulation at T, 1$\frac{1}{2}$T and 2T amplitudes (*p* < 0.001, random permutation test). Overall, the desynchronized state increased the early response for weaker stimuli (≤T) and the late response for the threshold and supra-threshold stimuli (≥T).

### Lower Response Variability in Desynchronized State

The efficiency of a neural code depends not only on the mean response produced for each stimulus, but also on the trial-to-trial variability. Here, we calculated Fano factor as a measure of trial-to-trial variability in evoked responses. Across all stimulus amplitudes, Fano factors were consistently lower in desynchronized state (Figure 4F) and this was not simply due to differences in mean firing rate across the two states (Figure 4G). The desynchronized state thus decreased the variability (increased the reliability) of neuronal responses.

### Higher Detection Performance in Desynchronized State

**Figure 5:**
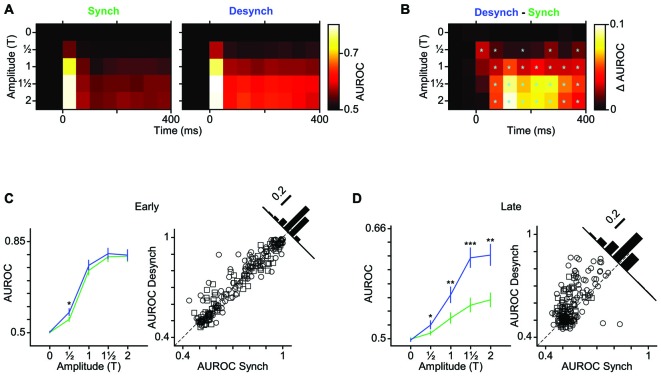
**Improved neuronal detection performance in desynchronized state. (A)** Neuronal detection performance is quantified as average area under ROC curves (AUROC) across units for each stimulus amplitude (*y*-axis) and at 50 ms intervals (*x*-axis) relative to the stimulus onset (0 ms). **(B)** Difference of AUROC between the two states. Boxes with cyan asterisk indicate significant differences (*p* < 0.05, random permutation test). **(C)** Average AUROC for the early response window (0–50 ms) across all units as a function of stimulus amplitude (left). Error bars are SEM. *Indicates *p* < 0.05, random permutation test. Right panel shows a scatter plot of the early response AUROCs for all units in the desynchronized vs. synchronized states. Squares represent single-units and circles represent multi-units. Inset histogram denotes the proportion of the AUROCs around the unity line (60% above and 39% below the unity line). **(D)** Same as **(C)** but for late response. Here, the six 50-ms windows during the late response phase (100–400 ms after stimulus onset) are averaged. Error bars are SEM across units. Inset histogram denotes the proportion of the AUROCs around the unity line (72% above and 28% below the unity line). ***p < 0.001, **p < 0.01, and *p < 0.05, random permutation test.

As demonstrated in the previous analyses, the desynchronized state increased the range of neuronal firing and reduced the trial-to-trial variability in neuronal response. This observation predicts a higher coding efficiency during the desynchronized state. We verified this prediction by performing an ROC analysis based on Signal Detection Theory (Green and Swets, 1966). To see the temporal dynamics of detection performance, we calculated the AUROC for consecutive 50-ms windows from 100 ms before to 400 ms after stimulus onset (Figure 5A). Difference in the AUROC between the two states confirmed our earlier prediction: detection performance was systematically higher in desynchronized state across almost all bins beyond 50 ms post stimulus onset (Figure 5B). For early responses (0–50 ms bin), the significant difference was observed only for the lowest stimulus amplitude ($\frac{1}{2}$T). Figures 5C,D further confirm this finding across all neuron-amplitude combinations. The desynchronized state significantly enhanced stimulus detectability at time instances beyond 100 ms (Figure 5D, random permutation test, *p* < 0.001).

### Reduced Response Latency in Desynchronized State

**Figure 6:**
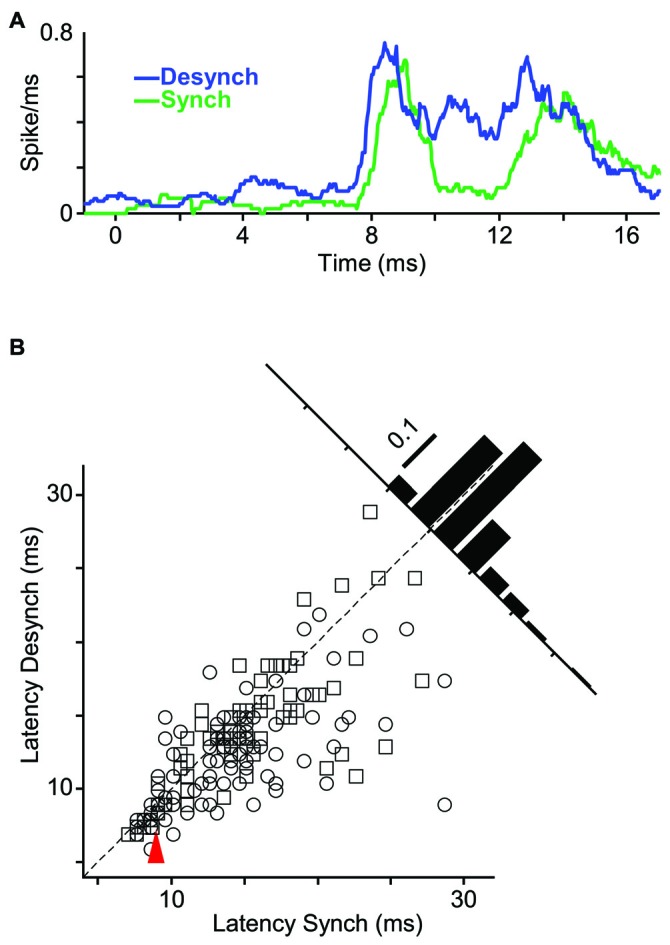
**Shorter response latency in desynchronized state. (A)** PSTHs of a representative multi-unit to whisker deflection of 1$\frac{1}{2}$T amplitude (18 μm; bin size, 1 ms; sliding at 0.5 ms steps). **(B)** Scatter plot represents latency of all single- and multi-units in desynchronized state vs. synchronized state. Inset histogram shows the distribution of response latency differences between the two states. Red arrow indicates the sample stimulus-unit in **(A)**.

Does state affect the temporal precision of spiking activity? We quantified this by measuring the response latency (as the first time bin that exceeded background activity by three standard deviations). Figure 6A shows sub-millisecond PSTH of an example unit. For this neuron, the response latency to an 18 μm (1$\frac{1}{2}$T) stimulus was 0.5 ms faster in the desynchronized state compared to the synchronized state. To examine the response latencies across all recordings, we focused on the neuron-stimulus pairs that produced an evoked response significantly above background activity in both states. Across all such neuron-stimulus pairs (Figure 6B; *n* = 171), the mean response latency was 12.9 ms in the desynchronized state and 14.4 ms in the synchronized state, and this difference was statistically significant (*p* < 0.001, random permutation test).

### LC Firing Rate as an Index of Cortical State

**Figure 7:**
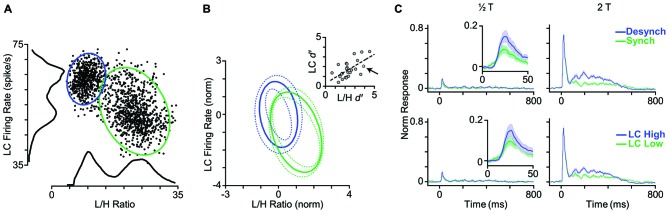
**LC firing rate identifies cortical state. (A)** Each dot represents one trial. LC firing rate and L/H ratio during a 5-s window centered on stimulus presentation. Histograms represent the corresponding distributions of L/H ratio and LC firing rate. Ellipses represent the standard deviation of the two states (synchronized in green, desynchronized in blue) based on a bivariate Gaussian mixture model. **(B)** Distribution of clusters averaged across 25 sessions. To combine sessions, Gaussian components were normalized to the distance between the two clusters. This normalization places the center of one of the Gaussian components on the origin (0, 0), and the other one on (1, −1). Inset: separation of clusters is quantified in each dimension using *d*^′^ measure. Every dot represents one session. The arrow indicates the sample session in **(A)**. The dashed line shows the linear fit to the data. **(C)** Top panel: the PSTHs are reproduced from Figure 4A where trials were categorized based on their L/H ratio. Bottom panel: trials are categorized based on the LC firing rate. PSTHs are normalized to the maximum spike per bin across amplitudes and states.

Given the strong and systematic correlation between L/H ratio and LC firing rate (Figure 2), we asked whether LC firing rate could replace L/H ratio and reliably indicate the cortical state. To address this question, we first measured the LC firing rate in a 5-s window centered on each whisker deflection stimulus (identical to the window used for the calculation of L/H ratio). Figure 7A shows the joint distribution of an example LC recording and the simultaneous L/H ratio for one representative session. Besides revealing the negative correlation between LC activity and L/H ratio, the distribution of LC activity across trials comprised two distinct peaks; each associated to a peak in the bimodal distribution of the L/H ratio. This pattern was systematically present across sessions (Figure 7B). In order to quantify the separation of trials into two distinct distributions, we fitted a Gaussian mixture model on the distribution of trials with two Gaussian components corresponding to the two cortical states (see “Materials and Methods” Section). This allowed us to calculate the overlap between the two clusters using the *d*^′^ index. We performed this quantification for the joint two-dimensional Gaussian fit parameters, and separately for each of the two dimensions (LC firing rate and L/H ratio) as well. The inset in Figure 7B reveals a remarkable correlation between the *d*^′^ values measured based on LC firing rate and those measured based on L/H ratio. Across sessions, the *d*^′^ based on LC firing rate was 1.47 ± 0.15 (mean ± SEM) while the *d*^′^ based on L/H ratio was 2.08 ± 0.18.

The final step was to categorize the response profile of BC units to whisker deflection based on the activity of the simultaneously recorded LC units. Similar to the state categorization based on the L/H ratio, we determined two thresholds according to the distribution of LC firing rate and categorized trials into two LC modes: LC-Low and LC-High. Figure 7C shows the average PSTH corresponding to each mode across all units. There was no prominent difference in response profiles between the two categorization methods (Figure 7C). Like L/H ratio, LC firing rate was predictive of sensory responses.

## Discussion

Here, we quantified the link between cortical state, spontaneous LC activity, and sensory processing in the rat BC. Under urethane anesthesia, we simultaneously recorded neuronal activity from BC and LC and determined cortical state by prefrontal EEG. We demonstrated that spontaneous fluctuations in LC firing rate preceded changes in the cortical state by an average of 1.2 s. We further characterized the state-driven modulations of sensory-evoked responses in cortical neurons. In the desynchronized state, BC neurons showed lower stimulus detection threshold, lower trial-to-trial variability, and shorter response latency.

The prominent state-driven change in BC response was observed during the late phase of evoked activity: the desynchronized state significantly increased the late response for almost every recorded BC unit. Recent evidence supports the role of late phases of neuronal response in sensory perception (Sachidhanandam et al., 2013; Crochet and Petersen, 2015). Reciprocal connections between the primary somatosensory cortex and the secondary motor areas of the prefrontal cortex provide the feedback which can modulate the late excitation in the somatosensory cortex (Manita et al., 2015). Rodent prefrontal cortex has the highest level of innervation from LC among all cortical areas (Loughlin et al., 1986; Sara and Bouret, 2012; Schwarz et al., 2015) and it provides reciprocal projection back to LC (Luppi et al., 1995). The prefrontal feedback to sensory cortices is thus a potential circuit through which LC firing could influence the late evoked activity and hence sensory perception.

Although cortical state changes along a continuum, two predominant modes are identified as the synchronized and desynchronized states (Harris and Thiele, 2011; Zagha and McCormick, 2014). These states alternate spontaneously during anesthesia (Clement et al., 2008; Marguet and Harris, 2011; Pachitariu et al., 2015), sleep cycles (Carter et al., 2010; Eschenko et al., 2012) and behavioral modes (Poulet and Petersen, 2008; Polack et al., 2013; Wekselblatt and Niell, 2015). The synchronized and desynchronized states observed under urethane anesthesia mimic the two states observed during natural sleep-wake cycles and the locomotion-induced desynchronization (Clement et al., 2008; Poulet and Petersen, 2008; Pagliardini et al., 2012; Polack et al., 2013). Some anesthetics predominantly induce one of the two states: Ketamine/xylazine anesthesia induces synchronized state (Hasenstaub et al., 2007; Pachitariu et al., 2015) while urethane/amphetamine (Bermudez Contreras et al., 2013) or fentanyl/medetomidine/midazolam (Pachitariu et al., 2015) predominantly induce desynchronized state.

Here, we identified the synchronized and desynchronized states based on the bimodal distribution of the L/H ratio (Figure 1E). The LC spiking activity also exhibited two distinct modes, which reflected the two modes of L/H ratio values (Figure 2). High LC firing rates coincided with low L/H ratios and hence were associated with the desynchronized state, while low LC firing rates coincided with high L/H ratios and hence were associated with the synchronized state. This link between LC activity and cortical state is compatible with studies demonstrating that LC activity fluctuates with the sleep-wake cycle (Aston-Jones and Bloom, 1981a; Eschenko et al., 2012) and level of arousal (Foote et al., 1980; Rajkowski et al., 1994). The fluctuations in LC firing rate preceded changes in cortical state. This is consistent with previous findings where perturbation of the LC-NE pathway altered the cortical state. For example, optogenetic stimulation of LC changed the level of arousal and increased the waking desynchronized states (Carter et al., 2010). Cortical application of NE suppressed slow-wave oscillation (Hirata and Castro-Alamancos, 2011; Castro-Alamancos and Gulati, 2014), and cortical application of NE blockers specifically disrupted desynchronized states in behaving animals (Constantinople and Bruno, 2011). Intraventricular injection of NE activated desynchronized EEG and increased the level of arousal (Matsuda, 1968, 1969; Cordeau et al., 1971) while systemic injection of NE antagonist caused a more synchronized EEG and induced sleep (Matsuda, 1968, 1969). Pharmacological or electrical activation of LC induced desynchronized state (Berridge and Foote, 1991; Marzo et al., 2014).

Fluctuations in pupil size are linked to cortical state in visual, auditory and somatosensory cortices (Reimer et al., 2014; McGinley et al., 2015a; Vinck et al., 2015). Given, the well-established role of LC-NE system in controlling pupil diameter (Rajkowski and Aston-Jones, 1993; Gilzenrat et al., 2010), the modulations in LC-NE system are likely to underpin the link between pupil diameter and the global brain state. Our findings further support the role of LC in cortical state fluctuation. However, activation of other brain regions such as thalamus (Hirata and Castro-Alamancos, 2010; Poulet et al., 2012; Eggermann et al., 2014), brain reticular formation (Castro-Alamancos, 2004; Hirata and Castro-Alamancos, 2011), cholinergic nuclei (Goard and Dan, 2009; Pinto et al., 2013; Lee et al., 2014), motor cortex (Zagha et al., 2013) and even single sensory neurons (Li et al., 2009) can also alter brain state. The causal interplay between neuromodulatory systems and brain state is thus complex and likely to involve serial or parallel activation/deactivation of cortical and subcortical areas.

In our data set, the proportion of neurons that were responsive to subthreshold stimuli increased from 7% in the synchronized state to 20% in the desynchronized state (Figure 3A). This specific increase in responsiveness has also been observed with LC microstimulation (Bouret and Sara, 2002). Cortical state is shown to modulate the evoked response in diverse ways. Some studies found response enhancement during desynchronized states in the visual (Niell and Stryker, 2010; Bennett et al., 2013; Polack et al., 2013; Lee et al., 2014) and olfactory (Murakami et al., 2005) cortices while others found response reduction during desynchronized states in the somatosensory cortex (Castro-Alamancos and Oldford, 2002; Castro-Alamancos, 2004; Crochet and Petersen, 2006; Zagha et al., 2013). This discrepancy suggests that the modulation of evoked response could depend on stimulus intensity. To test this, we selected stimulus intensities based on each neuron’s response threshold aligning them to critical positions along the stimulus-response function. This revealed that the modulation of the early response by state depended on stimulus intensity (see Figure 4B). Overall, more units showed higher response in the desynchronized state for T and this trend was reversed for the 2T (Figure 4B). At low stimulus intensities, BC neurons exhibit an accelerating non-linearity in their stimulus-response function, and this accurately predicts the detection and discrimination performance of rats (Adibi and Arabzadeh, 2011). Through its stimulus specific effect, the state can modulate the nonlinearity of the response function of cortical neurons and this in turn can adjust the detection and discrimination of stimuli based on the environmental context or behavioral demands.

We found that BC neurons exhibited lower Fano factors during the desynchronized state (Figures 4F,G). This is consistent with previous research showing that desynchronized states increase reliability in responses in somatosensory (Hirata and Castro-Alamancos, 2011; Zagha et al., 2013), visual (Goard and Dan, 2009; Reimer et al., 2014; Schölvinck et al., 2015) and auditory (Pachitariu et al., 2015) cortices. This reduced trial-to-trial variability is attributed to the lower variability of membrane potential in the desynchronized state (Poulet and Petersen, 2008; Polack et al., 2013; Reimer et al., 2014) as the up-and-down fluctuations during the synchronized state have a profound effect on neuronal responsiveness (Petersen et al., 2003; Hasenstaub et al., 2007; Safaai et al., 2015).

**Figure 8:**
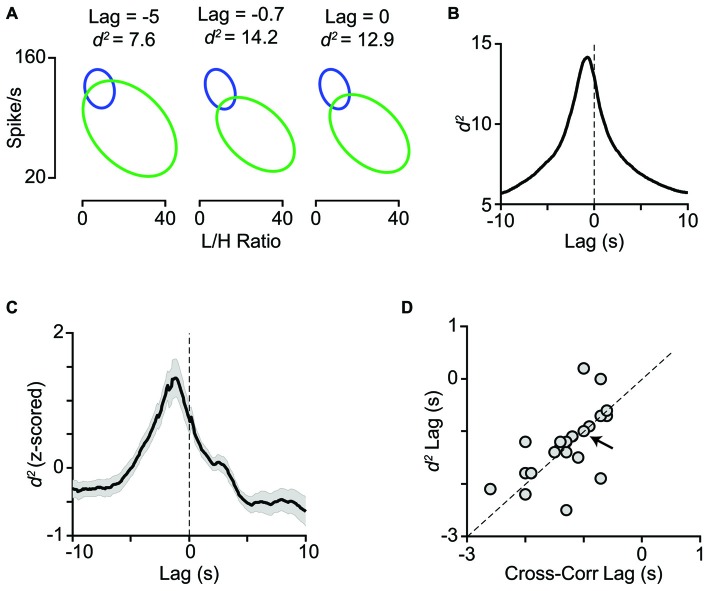
**Temporal relation between LC activity and cortical state. (A)** The joint distribution of LC firing rates and L/H ratios quantified with the bivariate Gaussian mixture model (as in Figure 7) obtained at three time lags (−5, −0.7 and 0 s). **(B)** For the same representative session as in A, *d^2^* is plotted as a function of lag between LC firing rate and L/H ratio. Maximum *d*^2^ was obtained at −0.7 s lag. **(C)** *Z*-scored *d*^2^ averaged across all sessions (*n* = 29). Maximum *d*^2^ was obtained at −1.2 s lag. **(D)** Maximum *d*^2^ lag is plotted against maximum cross-correlation lag for the sessions with negative peak in their cross-correlogram (21 sessions). The arrow indicates the sample session in **(A)** and **(B)** and the dashed line is unity.

Activation of the LC-NE system is shown to result in a range of neuronal response modulations, compatible with our findings. These include reduced temporal variability (Bouret and Sara, 2002; Lecas, 2004), mixed gain effects (Bouret and Sara, 2002; Berridge and Waterhouse, 2003; Devilbiss and Waterhouse, 2004, 2011; Devilbiss et al., 2006), reduced response latency (Lecas, 2004), changes in response threshold, and neuronal synchrony (Bouret and Sara, 2002). Activation of LC in a physiological range increases the level of extracellular NE in the cortical areas (Florin-Lechner et al., 1996). This elevation of NE is shown to alter sensory processing (McCormick, 1989; McCormick et al., 1991; Berridge and Waterhouse, 2003). The modulatory effects of NE resemble the state-dependent modulations of neuronal activity (Harris and Thiele, 2011; Pachitariu et al., 2015). Our analyses revealed a high degree of similarity between the trial categorization based on LC activity and based on L/H ratio (Figure 7). Furthermore, combining LC activity with the simultaneous EEG L/H ratio provided a better separation between the two cortical states. For the example session in Figures 8A,B, the highest separation was achieved when we combined L/H ratio with the preceding LC activity (at 0.7s earlier). Across all sessions, the highest separation was achieved when we combined L/H ratio with the preceding level of LC activity at 1.2s earlier (Figure 8C). The lag that produced maximum separation was consistent with the profile of cross-correlation between LC activity and L/H ratio (Figure 8D) and further confirmed the temporal relation between LC activity and state changes revealed by the earlier analysis (Figures 2C,D). The temporal relation between LC activity and changes in cortical state might depend on the type of anesthetic used. Future experiments could quantify this temporal relation under different anesthetics and in awake behaving animals.

The central nervous system’s ability to efficiently extract relevant information from the sensory environment is essential for survival. The whisker system is one of the main channels through which rodents collect information from their environment (Diamond and Arabzadeh, 2013). Behavioral studies have revealed a tight connection between neuronal activity in the BC and whisker mediated behavior (von Heimendahl et al., 2007; O’Connor et al., 2010, 2013). These behavioral studies typically use well-trained animals with reliable levels of performance. The degree of synchronization in cortical cells is shown to depend on the level of training: naïve animals elicit a more synchronized cortical state compared to trained animals (Sachidhanandam et al., 2013). The improved performance observed with training may be due to the LC-NE modulation of the cortical state. Consistent with this idea, the activity of the LC-NE system is shown to predict the behavioral performance of primates (Aston-Jones et al., 1999; Aston-Jones and Cohen, 2005). Non-noxious sensory stimuli can evoke phasic increases in LC neuronal activity in awake animals, indicating a potential role in sensory processing (Aston-Jones and Bloom, 1981b). However, LC neurons did not elicit an evoked response to the range of whisker deflections applied here, which might be due to the effect of anesthesia (Aston-Jones and Bloom, 1981b).

There has been substantial progress in understanding how BC neurons represent aspects of the animal’s environment such as object location (Knutsen et al., 2006; Knutsen and Ahissar, 2009; O’Connor et al., 2010), surface texture (Arabzadeh et al., 2005; von Heimendahl et al., 2007; Diamond et al., 2008; Wolfe et al., 2008) and whisker vibrations (Arabzadeh et al., 2003, 2004; Gerdjikov et al., 2010; Musall et al., 2014). Our results demonstrate a systematic relation between LC activity and the coding efficiency of vibrations in BC neurons. Chronic recordings from LC or perturbation of its activity during specific phases of the behavior (e.g., vibration presentation) could test its potential role in the reliable performances observed in whisker mediated sensory detection and discrimination tasks (Adibi et al., 2012; Mayrhofer et al., 2013; Fassihi et al., 2014; McDonald et al., 2014). Our findings support the involvement of the LC NE neuromodulatory system in the desynchronization of cortical state and the enhanced representation of the stimulus attributes.

## Author Contributions

ZF, YR-S, and EA designed research; ZF and YR-S performed experiments; ZF analyzed the data; ZF, YR-S, MA, and EA interpreted results of experiments; ZF prepared figures; ZF drafted manuscript; ZF, YR-S, MA, and EA edited and revised manuscript.

## Funding

Supported by an Australian Research Council (ARC) Discovery Project (EA; DP130101364) the ARC Centre of Excellence for Integrative Brain Function (CE140100007). ZF is supported by Iran’s National Elites Foundation and an IBRO-APRC Exchange Fellowship 2015. MA is supported by an NHMRC Early Career Fellowship, and EA is supported by an ARC Future Fellowship.

## Conflict of Interest Statement

The authors declare that the research was conducted in the absence of any commercial or financial relationships that could be construed as a potential conflict of interest.
